# Assessing a risk tailored intervention to prevent disabling low back pain - protocol of a cluster randomized controlled trial

**DOI:** 10.1186/1471-2474-11-5

**Published:** 2010-01-05

**Authors:** Carsten Oliver Schmidt, Jean-François Chenot, Michael Pfingsten, Ruth Anja Fahland, Gabriele Lindena, Ulf Marnitz, Klaus Pfeifer, Thomas Kohlmann

**Affiliations:** 1 Institut for Community Medicine, University of Greifswald, Walther Rathenau 48, D-17489 Greifswald, Germany; 2 Department of General Practice, University of Göttingen, Humboldtallee 38, D-37073 Göttingen, Germany; 3 Pain Clinic, University of Göttingen Robert-Koch Str. 40, D-37075 Göttingen, Germany; 4 Clara Institute, Clara Zetkin Str. 34, D-14532 Kleinmachnow, Germany; 5 Markgrafenpark Pain Center, Markgrafenstr. 19, D-10969 Berlin, Germany; 6 Institute of Sport Science and Sport, University of Erlangen Nuremberg, Gebertstr. 123 b, D-91508 Erlangen, Germany

## Abstract

**Background:**

Although most patients with low back pain (LBP) recover within a few weeks a significant proportion has recurrent episodes or will develop chronic low back pain. Several mainly psychosocial risk factors for developing chronic LBP have been identified. However, effects of preventive interventions aiming at behavioural risk factors and unfavourable cognitions have yielded inconsistent results. Risk tailored interventions may provide a cost efficient and effective means to take systematic account of the individual risk factors but evidence is lacking.

**Methods/Design:**

This study will be a cluster-randomised controlled trial comparing screening and a subsequent risk tailored intervention for patients with low back pain to prevent chronic low back pain compared to treatment as usual in primary care. A total of 600 patients from 20 practices in each study arm will be recruited in Berlin and Goettingen. The intervention comprises the following elements: Patients will be assigned to one of four risk groups based on a screening questionnaire. Subsequently they receive an educational intervention including information and counselling tailored to the risk group. A telephone/email consulting service for back pain related problems are offered independent of risk group assignment. The primary outcomes will be functional capacity and sick leave.

**Discussion:**

This trial will evaluate the effectiveness of screening for risk factors for chronic low back pain followed by a risk tailored intervention to prevent chronic low back pain. This trial will contribute new evidence regarding the flexible use of individual physical and psychosocial risk factors in general practice.

**Trial registration:**

ISRCTN 68205910

## Background

Low back pain (LBP) is an epidemiologically and economically important health problem [1-4]. An underlying specific pathology cannot be identified in most patients consulting in primary care and about 90% of all low back problems are therefore considered as being of non-specific origin [5]. Although it is expected that patients seen in primary care will recover within a few weeks a substantial proportion continues to suffer from LBP [6,7]. Therefore it is of high relevance to recognize patient characteristics predictive of a chronic or recurrent course of low back pain at an early stage [8]. Accordingly, a wide range of risk factors have been associated with the development and persistence of low back pain including life style factors, previous pain symptoms, psychosocial factors, work place factors, and sociodemographic as well as socioeconomic variables [9]. Among these, two sets of risk factors have demonstrated a particular importance in predicting the course of low back pain. Previous pain episodes [10-12], and psychosocial risk factors such as depression, and fear-avoidance beliefs [13,14].

Recent studies indicate that brief self-rating questionnaire based risk factor assessments may predict the course of low back pain and major related outcomes like sick leave [15-17]. Implementing a screening questionnaire might improve risk factor assessments in ambulatory settings. However, uncertainty remains how to translate the results of risk factor assessments into therapeutic action under field conditions. This concerns individual benefits as well as cost-effectiveness.

As could be demonstrated, educational interventions may be effective, in particular with regard to patients with acute or subacute back pain [18]. However, a brief provision of back pain related information may not be enough to substantially improve main indicators of disability such as sick leave [19]. Accordingly, there is no related recommendation in existing guidelines [20]. Information should rather be complemented with concrete cognitive-behavioural interventions [21,22].

While the European Guidelines on the treatment of acute back pain [20] conclude that scientific evidence does not support exercise therapy in patients with acute back pain they do agree on the benefits of the advice to stay active. Activating interventions may rather exert beneficial effects through an implicit change of adverse cognitions like fear-avoidance beliefs [23,24]. They may be more effective in patients with moderate disability and fear avoidance levels [25].

Content and extent of interventions should not be independent of the individual risk factors [25-27]. This may also be a reason for the limited effectiveness of recent trials on psychosocial interventions for back pain in primary care [28,29]. While risk tailored interventions have repeatedly been researched in other health related domains like smoking or hypertension [30-33] limited evidence is available with regard to pain problems, despite the repeated focus on subgroups [15,34,35]. Two studies evidenced the strength of tailored interventions with regard to musculoskeletal pain, and to a lesser degree temporomandibular disorders [36,37]. However there is a lack of evidence with regard to low back pain, in particular in combination with a screening tool that is easily implemented in general practice. Therefore we designed this randomized controlled trial to assess the effectiveness of a risk tailored-intervention program based on a brief risk screening. The study will be conducted in a primary care setting because this seemed most appropriate to target back pain patients at an early stage in the course of their illness.

## Methods/Design

### Aims

The aim of this study is to assess the effectiveness of a brief risk factor screening followed by a risk tailored intervention for patients consulting for acute to subacute low back pain in primary care. Key outcomes comprise functional capacity, sick leave, self-management activities, negative pain-related cognitions and a reduced utilization of health care services. Furthermore we aim to assess the acceptance of a short risk tailored-intervention among patients with acute and subacute low back pain and the utilization of an additional E-mail or telephone service.

### Design

**Figure 1:**
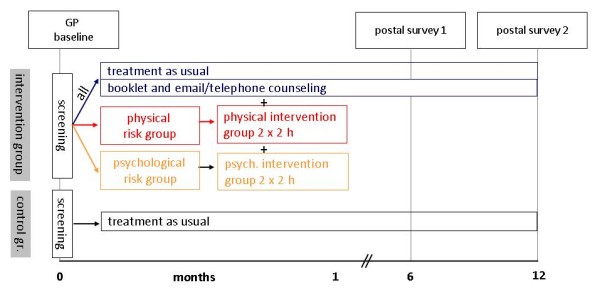
**Back pain and related variables recorded at baseline and after 6 and 12 months**.

The study is a two-armed cluster-randomized controlled study. General practices will be assigned at random to the control or intervention arm of the study and subsequently recruit patients. This cluster randomization approach is frequently used in ambulatory settings because of its high degree of internal validity and for pragmatic reasons[38]. Twenty GPs shall participate in each study region of whom half will be assigned to the control or the intervention group. Each GP should recruit 30 patients. This will lead to a total of 1200 patients with 600 belonging to the intervention, and 600 to the control arm of the study, respectively. Back pain and related variables will be recorded at baseline and after 6 and 12 months as outlined in Figure 1.

This sample size is based on a power analysis that accounted for an assumed drop-out of one third of the initial participants to the second follow up as well as for a loss of power due to correlated data within primary sampling units. Based on previous results in a GP intervention study [39], an intraclass-correlation of 0.025 is a plausible assumption for the target outcomes of our study. Given a final cluster size of on average 20 subjects the design effect will be approximately 1.5. To detect a difference in functional disability between intervention and control group at the second follow-up that corresponds to a Cohen's d effect size of 0.3 with a significance level of 5%, and a power of 80%, a final sample of 265 subjects in each study arm will be necessary. Assuming a total drop out rate of even 40% till the second and last follow up, the initial sample size of 600 subjects per study arm will suffice to detect the outlined effect.

### Study population and recruitment

#### General practitioners

General Practitioners (GPs) in Göttingen and vicinity and Berlin are invited to take part in the trial by letter. Addresses are obtained from the local health boards. In case of non-response to the letter GPs are contacted by telephone or personally. From previous experience it is known that roughly 15-20% agree to participate [40].

#### Patients

Consecutive patients consulting for low back pain in general practices will be invited to participate in the study. Practice nurses will perform a first screening for eligibility based on non-medical criteria. Patients will receive an information leaflet regarding the purpose and content of the study. Patients willing to participate complete the consent form. A list of inclusion and exclusion criteria is given in Table 1. Should any of the exclusion criteria be recognized during the subsequent consultation, the patient will be informed by the doctor and excluded from the study.

**Table 1:** Inclusion and exclusion criteria

Inclusion criteria	• consulting for low back pain
	• age 20 to 60 years
	• German language proficiency
	• ability to give informed consent
Exclusion criteria	• treatment for the present low back pain episode for more than 3 months
	• severe co-morbidity (cardiovascular, metabolic, inflammatory disease or tumors)
	• back pain of specific origin, e.g. ankylosing spondylitis
	• prior spine surgery
	• specialized pain treatment or rehabilitation for low back pain within the last 5 years
	• ongoing early retirement or pension claim
	• poor general condition
	• severe pain during night or rest
	• signs of radiculopathy like numbness, muscle weakness, loss of reflexes

### Randomization

The randomization of GPs to the intervention or control arm will be conducted externally by the coordinating centre of the study in Greifswald. GPs consenting to participate are reported by the two regional coordinators to the study centre. The subsequent randomization will be conducted blockwise with blocks of four, six, and eight units length, to veil the possible allocation of the next GP.

### Ethics

The study is planned and conducted in accordance with medical professional codex and the Helsinki Declaration of 1996 as well as the German Federal Data Security Law (BDSG). Patients participate voluntarily. The study protocol was approved by the ethics committee of the University of Greifswald and Göttingen prior to the start of the study in March 2009. Patients receive written and spoken information about the main features of the study; i.e. about potential benefits for their health and potential risks prior to their consent and participation in the study. In case of acceptance, they sign the informed consent sheet. Patients are informed that they may cancel their participation at any time without disclosing reasons and without negative consequences to their medical care.

### Intervention

The intervention consists of the following elements: Firstly, a risk-factor screening, secondly, a risk tailored information and counselling, and thirdly a telephone/email consulting service for back pain related problems independent of risk group assignment. These elements are described subsequently:

#### Risk screening

After giving their informed and written consent to participate in the study patients will receive a brief self-rating risk screening inventory concerning yellow flags that are predictive of chronic back pain. The screening items are based on a German translation of the Örebro Musculoskeletal Pain Screening Questionnaire [41,42]. Ten out of the 25 items comprised by the instrument were selected for our investigation: pain intensity in the past week (one item), fear-avoidance beliefs (two items), depressive mood (two items), functional ability (four items), and body locations of pain (one item). The selection of these items was mainly based on previous findings concerning predictors for the course of back pain as outlined in the introduction. A brief instrument based on the selected domains was useful in predicting disability [15] and provided the conceptual background for our risk groups as described below.

The completed questionnaires will be returned to the practice nurse prior to the consultation and immediately analyzed to be available during consultation only in the intervention arm. Based on their screening responses patients in the intervention arm will be assigned to one of four risk groups (low/only physical/only psychological/physical and psychological). The low risk group comprises subjects without strong pain symptoms (defined by high intensity low back pain with functional limitations or multilocated pain) as well as low levels of depression and fear avoidance. The physical risk group comprises subjects with severe low back pain. Subjects that belong to this group experience high intensity and disabling back pain as well as pain in different body locations. The psychological risk group comprises subjects with strong low back pain and high levels of depression and fear avoidance. Subjects may be assigned to either one or both risk groups.

Apart from the subsequently described risk tailored intervention all patients receive treatment as usual. A national guideline for management of low back pain in primary care was published in 2001 [20].

#### Risk tailored intervention

The tailored interventions are based on the risk factor assignment.

#### Low risk group

Patients receive advice on staying active by the general practitioner and will be handed the back book. This is a German translation of the educational booklet developed by Burton et al [43]. This booklet fosters health related behaviours and was effective in reducing erroneous back related beliefs and fosters active health related behaviours. It argues against a biomechanical model by emphasizing psychological and social aspects of back problems. In addition patients receive additional information on local activities on physical exercise, sports and relaxation.

#### Physical risk group

In addition to the physicians' advices and the back book patients assigned to this group will be offered participation in a guideline based intervention group that consists of two meetings of 120 minutes duration each. The main focus of these manualized meetings will be: education on "back myths" and risk factors, introduction to self controlled exercise, and strategies to become and stay active. The groups will be conducted by experienced exercise therapists. The group size is limited to ten patients.

#### Psychological risk group

In addition to the physicians' advices and the back book patients assigned to this risk group will be invited to participate in a psychosocial risk factor group that comprises two additional meetings of 120 minutes duration each. These meetings will be conducted by psychologists with a specialization in pain treatment. Patients receive a manualized cognitive-behavioural intervention that focuses on pain and strain prone situations as well as on catastrophizing, depressive, and fear-avoidant cognitions. The group size is limited to six patients.

To monitor compliance an attendance list will be kept for every course. Patients receive an illustrated coloured handbook designed specifically for the purpose of this trial with information on all covered topics. All therapeutic sessions will be structured based on a written manual. All participating therapists will have attended a training session of four hours.

#### Telephone, email counselling

The aim of this counselling offer is to answer back pain related questions that usually cannot be handled during a normal consultation. Local study coordinators (usually physiotherapists) will take on enquiries and refer them to the collaborating physicians, psychologists, and sport scientists, as best suited. No diagnoses will be made during these contacts; neither will there be concrete therapeutic recommendations. A manual is authored to standardize responses given during the telephone/email counselling.

### Control group

Control patients will receive treatment as usual. They participate in the accompanying survey, including the baseline assessment, and the two follow-up surveys at six and twelve months. Furthermore, control patients complete the risk screening prior to their consultation. However, results of this screening will not be communicated to the doctor nor to the patient. Because control patients are recruited in different practices from intervention patients there is a low risk of cross-contamination.

### Hypothesis

Primary hypothesis: The risk tailored intervention is more effective in reducing disability (functional disability and sick leave) compared to treatment as usual.

Furthermore we hypothesize that a risk tailored intervention should promote self-management activities, lead to fewer negative pain-related cognitions and reduce the use of health care services because of non-specific back pain.

### Assessing Selection Bias

Non-response bias may threaten the validity of our results. Therefore all practices will document successful and failed recruitment attempts. Age and sex of patients who refuse to participate will be recorded. Drop-out to the follow up surveys will be statistically controlled based on the participants' responses at baseline as described in data analysis.

### Primary Outcomes

Two primary outcomes were chosen with a high importance for patients as well as for health care providers: Functional disability as measured with the Hanover Functional Ability Questionnaire [44] and days of sick at the second follow up after 12 months. The latter is in particular important for a monetary assessment of the benefits of the brief intervention program.

### Secondary Outcomes

A list of secondary outcomes is depicted in Table 2. Measures comprise the severity of back pain, depression, quality of life, fear-avoidance, catastrophizing, physical activities, and health care utilization. Effects of the intervention after 6 months and 12 months will be compared.

**Table 2:** Outcomes and Instruments

Sociodemographic data	Deck R, Röckelein E. DRV-Schriften 1999, 16: 84-102. K.-H. Jöckel, B. Babitsch, et al. 1998, in: W. Ahrens et al. München: MMV Medizin Verlag. 7-38
Pain intensity, disability	Graded Chronic Pain Scale; Von Korff M, Ormel J, Keefe FJ, Dworkin SF. Pain 1992, 50: 133-49.
Functional capacity	FFbH -R; Kohlmann Th, Raspe H. Rehab 1996, 35: I-VIII.
Quality of life	SF 12; Gandek et al. J Clin Epidemiol. 1998, 51:1171-8
Use of medical services for LBP	Core set items of the German Back Pain Research Network
Depression	PHQ-D; Gräfe K, Zipfel S, Herzog W, Löwe B. Diagnostica 2004, 50(4):171-181.
Fear avoidance beliefs	FABQ; Pfingsten M, Kröner-Herwig B, Leibing E et al. European Journal of Pain 2000, 4: 259-266.
Physical activity, use of health promoting services	Wagner, P, Singer, R (2003). Sportwissenschaft 33 (4), 383-397.
Catastrophising	FSS; Flor, H., Behle, D. B., und Birbaumer, N. (1993). Behaviour Research and Therapy, 31, 63-73.

Additionally, participation in the counselling groups and telephone/email contacts will be documented.

### Data analysis

Baseline and follow up data will be presented using common parametric and non-parametric descriptive statistics. In addition to cross-sectional analysis to compare the control and intervention group at the second and last follow up, longitudinal analysis will use multilevel regression and generalized estimation equating (GEE) models with the outcome variables as outlined above. Non-response and drop out will primarily be accounted for by calculating statistical weights, or by conducting complete case analysis under the missing at random assumption, as appropriate. Comparisons predominantly concern the entire intervention group vs. the control group. A time vs. treatment group interaction will be computed to assess the benefits of the brief risk tailored assessment vs. treatment as usual.

### Data safety and privacy

Patient names and other confidential information are secured by the medical confidentiality rules and treated according to German Federal Data Security Law. Informed consent and questionnaires are mailed to study centres independent of each other to disable the tracking of individual patients.

## Discussion

In this paper we present rationale and design of a cluster randomized controlled trial to evaluate a risk tailored brief intervention program to prevent chronic low back pain. It will provide evidence concerning the usefulness of a screening questionnaire to guide treatment of patients with acute and subacute low back pain in general practice and if two, respectively four sessions are effective to reduce the risk of persisting back pain, and related disabilities. If it is possible to install a short conclusive questionnaire into routine screening it will be cost and time efficient at the long run.

The results of this research will be presented as soon as they are available.

## Abbreviations

The following abbreviations have been used in the manuscript: GP: General practitioners; LBP: low back pain.

## Competing interests

The authors declare that they have no competing interests.

## Authors' contributions

All authors contributed to the study design. COS and JFC were the principal authors of the manuscript. All authors contributed to manuscript drafting and revision and approved the final manuscript.

## Pre-publication history

The pre-publication history for this paper can be accessed here:

http://www.biomedcentral.com/1471-2474/11/5/prepub
